# Acute kidney injury in children with chronic liver disease

**DOI:** 10.1007/s00467-018-3893-7

**Published:** 2018-03-01

**Authors:** Akash Deep, Romit Saxena, Bipin Jose

**Affiliations:** Paediatric Intensive Care Unit (PICU), King’s College Hospital, Denmark Hill, London, SE5 9RS UK

**Keywords:** Acute kidney injury, Liver failure, Hepatorenal syndrome, Cirrhosis, Biomarkers

## Abstract

Acute kidney injury (AKI) is a common accompaniment in patients with liver disease. The causes, risk factors, manifestations and management of AKI in these patients vary according to the liver disease in question (acute liver failure, acute-on-chronic liver failure, post-liver transplantation or metabolic liver disease). There are multiple causes of AKI in patients with liver disease—pre-renal, acute tubular necrosis, post-renal, drug-induced renal failure and hepatorenal syndrome (HRS). Definitions of AKI in liver failure are periodically revised and updated, but pediatric definitions have still to see the light of the day. As our understanding of the pathophysiology of liver disease and renal involvement improves, treatment modalities have become more advanced and rationalized. Treatment includes reversing precipitating factors, such as infections and gastrointestinal bleeding, volume expansion, paracentesis and vasoconstrictors. This approach is tried and tested in adults. A pediatric tailored approach is still lacking due to inadequate numbers of patients, differences in causes of AKI and paucity of literature. In this review, we attempt to explore the pathophysiological basis, treatment modalities and controversies in the diagnosis and treatment of AKI in pediatric patients with chronic liver disease and discuss our own personal practice. We recognize that, although it is not a very commonly encountered entity in pediatric population, HRS has specific diagnostic criteria and treatment modalities that differ from other causes of AKI in patients with chronic liver disease; hence among the etiologies of kidney injury in patients with chronic liver disease, we focus here on HRS.

## Introduction

The association between liver disease and renal failure has been known for over a century. In the 1800s, Flint and co-workers performed extensive work on this association of kidney involvement in patients with liver disease [1]. Renal involvement in childhood liver diseases is not unusual in the context of genetic conditions with multisystem involvement, such as Alagille syndrome [2, 3] and metabolic disorders either related to an enzymatic defect in the liver causing renal impairment, such as primary hyperoxaluria, or other conditions, such as organic acidurias, urea cycle disorders or glycogen storage disorders, among others. [4, 5]. However, these are known associations of renal involvement in patients with liver disease. Kidney involvement in those liver diseases where kidney injury is not present initially but takes place in an acute setting is a completely different entity. Though acute kidney injury (AKI) has been extensively studied in patients with non-liver conditions, it is only recently that clinicians have started to explore the importance of AKI in patients with liver disease. The incidence and prognosis of AKI in patients with liver disease varies depending on the liver disease in question—acute liver failure, chronic liver disease, acute-on-chronic liver failure (ACLF), post-liver transplantation or metabolic liver disease. AKI is associated with a tenfold increase in mortality in patients admitted to critical care [6]. It has been observed that approximately 20% of patients hospitalized with cirrhosis develop AKI [7]. In this review, we explore some of these issues and current dilemmas in the diagnosis and management of AKI in liver failure, especially in patients with chronic liver disease. We would like to emphasize that there is an extreme paucity of data in the pediatric literature, and most of the concepts are extrapolations from the adult literature.

## The natural history of chronic liver disease

Most affected children develop end-stage liver disease secondary to chronic liver disease that originated during infancy. The most important underlying disease in these children is biliary atresia. Though there are many similarities between the pathophysiology of chronic liver disease in children and adults, there are important differences as well, the most notable being failure to thrive in children, which includes the inability to gain adequate weight and linear growth, nutritional deficiencies (e.g. fat-soluble vitamins) and impairment of developmental growth.

**Fig. 1:**
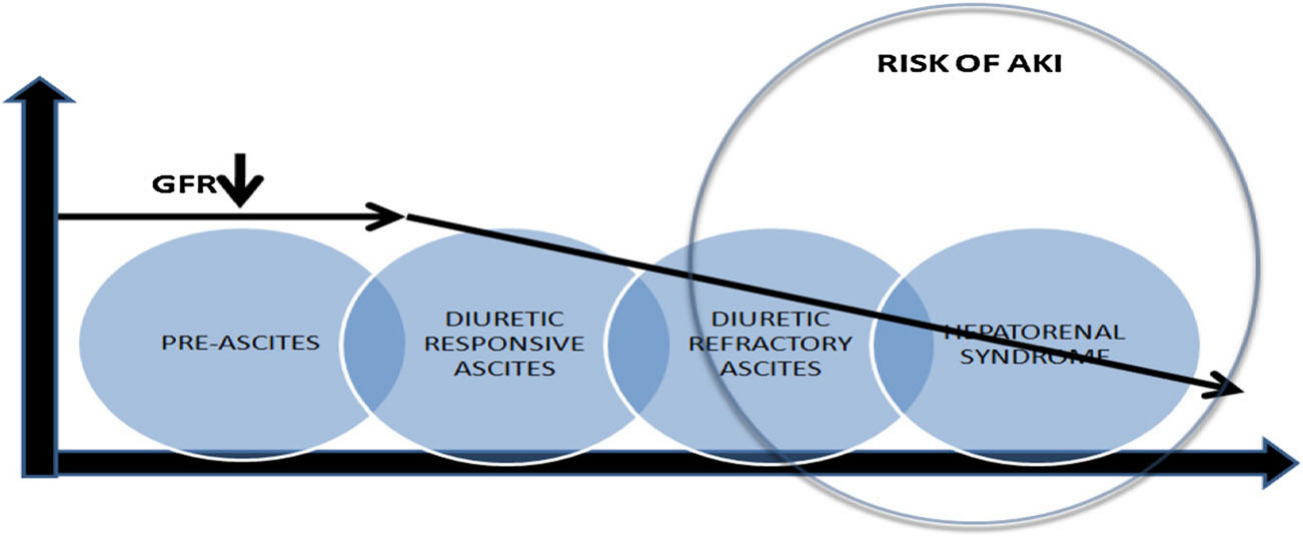
Natural history of acute kidney injury in liver disease.* GFR* Glomerular filtration rate, *AKI* acute kidney injury

Before we discuss AKI in patients with chronic liver disease, it is vital to understand the progression of liver disease in these patients. The natural history of cirrhosis passes through two stages—an asymptomatic phase which is well compensated and usually followed by a progressive phase characterized by the development of complications of portal hypertension and/or synthetic liver failure. Here we need to differentiate between a cirrhotic patient whose underlying liver disease worsens accompanied by the development of complications, including portal hypertension and renal dysfunction, as opposed to a stable patient with chronic liver disease with reasonable liver reserve who gets hit by a precipitating event like bleeding, infection or volume loss, which converts a ward level patient into a high-intensity patient with multi-organ failure, including renal involvement (Fig. 1). In both scenarios, kidney involvement can take place, though the prognosis will be different.

## AKI in liver disease—definitions

The diagnosis of AKI in patients with non-liver disease using the pRIFLE (Pediatric Risk, Injury, Failure, Loss of function and End-stage renal disease), AKIN (Acute Kidney Injury Network) or KDIGO (Kidney Disease for Improving Global Outcomes) criteria is based on two parameters, namely the absolute value of serum creatinine (SCr) or percentage change from baseline SCr plus urine output. These two parameters form the basis of diagnosis and eventual staging of severity [8–10]. Unfortunately, the AKI definitions used in patients with cirrhosis have not been very well developed and lack standardization and sensitivity [11, 12].

There is a problem with both of these parameters in patients with cirrhosis. SCr is affected by two important factors, muscle mass and liver synthetic function, both of which are impaired in advanced liver disease. Baseline SCr is significantly lower in patients with cirrhosis due to protein malnutrition and muscle wasting [13–16]. In addition, changes in SCr are small and delayed for any given change in glomerular filtration rate (GFR), resulting in an underestimation and, unfortunately, impairing the recognition of changes in GFR in patients with cirrhosis [17, 18]. Therefore, more than 50% of glomerular function may be lost before the diagnosis of AKI is considered [19, 20], possibly delaying timely access to AKI management and adversely affecting prognosis. In addition, bilirubin is known to interfere with assays, where hyperbilirubinemia masks rises in SCr [17].

The use of oliguria as a diagnostic criterion for AKI in patients with cirrhosis is also flawed. These patients might have decreased urine output, yet may have relatively normal GFR. Urine output might also be decreased because of intra-abdominal hypertension secondary to massive ascites. On the other hand, urine output may be artificially increased with the use of diuretics. Therefore, decreased or increased urine output in patients with cirrhosis must be interpreted with caution. Thus, despite all its drawbacks, kinetic changes in SCr are used as the single most important parameter in the definition of diagnosis of AKI in patients with cirrhosis [12, 21].

In view of these difficulties in defining AKI in patients with liver disease, the second International Consensus Conference of the Acute Dialysis Quality Initiative (ADQI) in 2010 defined the term “hepatorenal disorders (HRD)” to describe patients with advanced cirrhosis and concomitant renal dysfunction. This definition allowed inclusion of any form of renal disease occurring concomitantly with cirrhosis [11]. The first important difference in these guidelines is that the urine output component (which is a key feature of AKI diagnostic criteria in non-liver disease patients) has been removed as a criterion to diagnose AKI in patients with liver disease [11, 12, 22]. Secondly, it is recommended that a delta increase in SCr from baseline is considered rather than the absolute value. Hence, the conventional criterion of a rise of SCr to reach an absolute value of 1.5 mg/dl to diagnose AKI in a patient with liver disease has been removed [12, 23]. These revised definitions are given in Table 1.

**Table 1:** Diagnosing and staging of acute kidney injury in liver failure [7, 8, 11, 12, 24, 25]

Revised definitions for the diagnosis and staging of AKI in liver failure
Baseline SCr: a value of SCr obtained in the previous 3 months, when available, can be used as baseline SCr. In patients with more than one value within the previous 3 months; the value closest to the admission time to the hospital should be used.
In patients without a previous SCr value, the SCr value on admission should be used as baseline. Definition of AKI: • Increase in SCr of ≥ 0.3 mg/dl (≥ 26.5 mmol/L) within 48 h; or • A percentage increase in SCr of ≥ 50% from baseline ,which is known, or presumed, to have occurred within the prior 7 days
No response: no regression of AKI Partial response: regression of AKI stage with a reduction of SCr to ≥ 0.3 mg/dl (26.5 μmoll/L) above the baseline value Full response: return of SCr to a value within 0.3 mg/dl (26.5 μmol/L) of the baseline value.
Staging of AKI (ICA-AKI criteria) • Stage 1: increase in SCr of ≥ 0.3 mg/dl (26.5 μmol/L) or an increase in SCr of ≥ 1.5- to 2-fold from baseline. • Stage 2: increase in SCr of > 2- to 3-fold from baseline • Stage 3: increase of SC of > 3-fold from baseline or SCr of ≥ 4.0 mg/dl (353.6 μmol/L) with an acute increase of ≥ 0.3 mg/dl (26.5 μmol/L) or initiation of renal replacement therapy

AKI, Acute kidney injury; SCr, serum creatinine; A ICA, International Club of Ascites

## Classification of kidney injury in liver failure

Acute kidney injury in liver failure can be multifactorial. The predominant causes of AKI in cirrhosis can be either functional or structural. Functional causes include pre-renal azotemia and hepatorenal syndrome (HRS), while structural causes are predominantly due to intrinsic renal disease which may be tubulo-interstitial or glomerular in origin [26]. The importance of etiopathological diagnosis was demonstrated by Martín-Llahí et al., who found that the most common causes of AKI in cirrhosis were pre-renal azotemia, acute tubular necrosis (ATN) and HRS [27]. Other studies found infection to be a predominant cause for AKI in patients with chronic liver disease (biliary or gastrointestinal tract infections, spontaneous bacterial peritonitis and urinary tract infection) [28, 29]. A study by Belcher et al. found that of the total 188 patients with cirrhosis, 53% had ATN, 26% had pre-renal azotemia and 22% were diagnosed with HRS [30]. The common causes of AKI in liver disease are enumerated in Table 2.

**Table 2:** Causes of acute kidney injury in liver failure [6, 7, 11, 12, 23–26, 28]

Causes of acute kidney injury (AKI) in liver failure
Functional causes
• Pre-renal azotemia: volume responsive states include shock, dehydration, sepsis, decreased intake of fluids, excessive gastrointestinal losses including diarrhea, vomiting and gastrointestinal bleeding
• HRS type 1 and 2: (volume unresponsive state): now called as HRS-AKI [12]
Structural causes
• Intrinsic renal disease as acute tubular necrosis (ATN), tubulo-interstitial and glomerular diseases
• Post-renal AKI—obstruction (Post-transplant AKI is a separate entity with multifactorial causation)

HRS, Hepatorenal syndrome; HRS-AKI, HRS type of AKI

## Kidney injury in ACLF

Acute-on-chronic liver failure is a specific syndrome characterized by acute decompensation (which may be due to hemorrhage, bacterial infection or massive paracentesis) in a patient with chronic liver disease. It is associated with organ failure and high short-term mortality (28-day mortality 29.7–33.9% vs. 1.9% among patients who do not develop ACLF) [31, 32]. In adults, the majority of patients who develop ACLF will have chronic liver disease of varying etiologies, whereas in the pediatric patient population obvious chronic liver disease is recognized only in a limited number of conditions, the majority of which include biliary atresia, Wilson’s disease and autoimmune liver disease. Triggers for precipitation of acute liver insufficiency will depend upon geographic location, timing of diagnosis and severity of the presentation.

The Chronic Liver Failure (CLIF) Consortium has proposed using the CLIF Consortium-Sequential Organ Failure Assessment (CLIF-SOFA) score at admission to screen all cirrhotic patients for the diagnosis of ACLF [33]. This score, unlike previous scores for liver patients, has kidney dysfunction at the heart of classifying patients for severity of liver failure and subsequent management and transplantation: the more severe the kidney injury, the poorer the prognosis. This score has been tested in adults but needs validation in the pediatric patient population. Alam et al. were able to demonstrate the application and reliability of the CLIF-SOFA score in a pediatric setting and its preference over other severity scores, including models for end-stage liver disease and pediatric end-stage liver disease [34].

## Hepatorenal syndrome

Hepatorenal syndrome is a type of AKI which was initially defined as a form of functional renal failure caused by intra-renal vasoconstriction that occurs in patients with end-stage liver disease and circulatory dysfunction. The definition and diagnostic criteria for HRS were originally established in 1994 [35].

The International Club of Ascites (ICA) has been convening periodically since 1994 to define HRS [12, 25, 35]. The definition of HRS is based on exclusion of other causes of AKI with concomitant unresponsiveness to volume expansion. The latest criteria to diagnose AKI, and specifically HRS, in liver disease were proposed by ICA in 2012 and are summarized in Table 3.

**Table 3:** Definitions of hepatorenal syndrome [12, 24, 36–42]

HRS definitions
HRS-AKI (ICA 2015 criteria) • Diagnosis of cirrhosis and ascites • Diagnosis of AKI according to ICA-AKI criteria as described in Table 1 • No response after 2 consecutive days of diuretic withdrawal and plasma volume expansion with albumin 1 g per kg of body weight • Absence of shock • No current or recent use of nephrotoxic drugs (NSAIDs, aminoglycosides, iodinated contrast media, etc.) • Emphasizes the use of urine biomarkers (as NGAL, KIM1, IL-18, FABP and albumin) in differentiating HRS and ATN • No macroscopic signs of structural kidney injury defined as: - absence of proteinuria (> 500 mg/day) - absence of microhematuria (> 50 RBCs per high power field), - normal findings on renal ultrasonography
Criteria for HRS in cirrhosis (as per ICA, 2007) [25]: • Cirrhosis with ascites. • SCr of >133 mmol/L (1.5 mg/dl) (this has been removed from the 2012 criteria) • No improvement of SCr (decrease to a level of ≤133 mmol/L) after at least 2 days with diuretic withdrawal and volume expansion with albumin (1 g/kg/day)(maximum of 100 g/day) • Absence of shock. • No current or recent treatment with nephrotoxic drugs. • Absence of parenchymal kidney disease as indicated by proteinuria of > 500 mg/day, microhaematuria (> 50 RBCs per high power field) and/or abnormal renal ultrasonography.

NSAIDs, Nonsteroidal anti-inflammatory drugs; NGAL, neutrophil gelatinase associated lipocalin; KIM1, kidney injury molecule 1; IL, interleukin; FABP, liver-type fatty acid-binding protein; ATN, acute tubular necrosis; RBCs,:red blood cells; HRS, hepato-renal syndrome; AKI, acute kidney injury; ICA, International Club of Ascites

It is important to know the practical differences which exist between the incidence of HRS in adult and pediatric populations. In the pediatric patient population, cirrhosis is mostly secondary to biliary atresia. Only 10% of patients have entirely normal liver function tests after Kasai porto-enterostomy, while 40% of patients will survive with their native liver after 10 years following Kasai operation [43]. Approximately 80% of patients who undergo Kasai surgery will ultimately need a liver transplant [44]. These children are kept under close follow-up. As cirrhosis, ascites and portal hypertension set in and ascites becomes diuretic resistant, these children are immediately listed for transplant. Since the number of children with such conditions is small, coupled with the advent of split liver transplant, waiting times are relatively short and the incidence of complications, including HRS, is not very high. In adult patients, etiologies are varied, numbers are high and waiting lists are extremely long. Therefore, adult patients develop all the complications of cirrhosis and portal hypertension, including HRS.

Although renal dysfunction and HRS have been occasionally reported in infants and children with end-stage liver disease, these reports are primarily case reports without any large series. Thus, the true frequency of HRS in pediatric patients with liver failure is currently unknown.

As per the ICA 2007 guidelines, HRS has been classified into two types, based on the rate of rise in SCr in association with cirrhosis [12, 24, 36]. Type-1 HRS (HRS-1) is defined as “doubling of the serum creatinine to a level greater than 2.5 mg/dL in less than 2 weeks’ duration.” Type-2 HRS (HRS-2), on the other hand, is defined as a gradual rise in SCr to > 1.5 mg/dl. The two types of HRS are treated as two different clinical entities rather than as stages of progression of the same disease. HRS-1 is more acute, is more commonly associated with multi-organ failure, has a very grim prognosis and overlaps with other causes of AKI. A precipitating event (sepsis, acute bleeding episode, massive paracentesis) is identified in 70–100% of HRS-1 patients, and more than one event can occur in a single patient.

HRS-2 is the true form of AKI in patients with cirrhosis. It is representative of the progressive, severe circulatory changes which take place in cirrhosis. As opposed to HRS-1, HRS-2 progresses slowly and its progression is directly proportional to the deterioration in liver function [24, 36]. The median survival time of HRS-1 is less than 2 weeks, and practically all patients die within 8–10 weeks after the onset of renal failure [37, 45].

Because HRS rarely occurs in children, there are no specific definitions in the pediatrics literature. Yousef et al. in 2010, published data on treating four children with HRS and used the ICA criteria except “doubling or more of the serum creatinine concentrations (from baseline) over a two-week period with no set cut-off values” [11, 12, 46]. However, with the removal of the cutoff of 1.5 or 2.5 mg/dl for the diagnosis of HRS in adults in the 2012 ICA meeting, it might be possible to extrapolate the adult definition in children akin to the RIFLE criteria. We believe that these criteria should be examined prospectively in a large group of children (in various liver units) with chronic liver disease to provide validation.

## Epidemiology of HRS

Though it is a common belief that whenever there is kidney involvement in patients with liver disease the diagnosis of HRS should be entertained, HRS is the least common diagnosis in these patients. The most common causes of AKI in these patients include pre-renal AKI and ATN. Hypovolemia is the commonest cause of AKI, possibly due to gastrointestinal hemorrhage secondary to variceal bleed or due to fluid losses either from the kidneys (secondary to excessive diuretic use) or gastrointestinal tract (excessive lactulose administration or gastrointestinal infection). Parenchymal renal disease is not very common in the pediatric patient population. Drug-induced kidney injury is not uncommon and should be vigilantly looked for.

While approximately 50% of cirrhotic patients with ascites will develop AKI during their illness, HRS constitutes only a small fraction of all AKI cases that develop in these patients. In an analysis of 129 adult cirrhotic patients with ascites and AKI, HRS was responsible for the deterioration of kidney function in only 7.6% [47].

HRS is usually a diagnosis of exclusion—all identifiable causes of renal failure need to be excluded before a diagnosis of HRS is made. The true incidence of HRS in pediatric patients is lacking due to the small number of patients associated with many confounding factors. In the pre-transplant era, the incidence of renal involvement in liver disease was estimated to be approximately 5%. However, it is also important to note that there are a variety of hepatic diseases requiring liver transplantation that have associated renal involvement not related to HRS, which may confound accurate incidence of HRS in children. These diseases include hereditary tyrosinemia type-I, autosomal recessive polycystic kidney disease, Alagille syndrome, primary hyperoxaluria and other genetic disorders.

## Pathophysiology of AKI in HRS

There are a number of theories to explain the pathophysiologic mechanisms for AKI in chronic liver disease, especially HRS, a few of which are discussed in this section.

### Portal hypertension, bacterial translocation and vasodilation

The triggering event is the presence and severity of portal hypertension. Organisms in the portal circulation are usually cleared by hepatocytes and Kupffer cells, which constitute the mononuclear–phagocyte system of the liver. As cirrhosis develops and portal hypertension sets in, the intestinal mucosa becomes swollen due to obstruction of drained portal blood. This, along with decreased intestinal movements, result in massive bacterial overgrowth in the intestinal lumen, especially of Gram-negative enteric organisms which produce endotoxins. Because of defects in mucosal barrier function and decrease in mononuclear–phagocyte cells, bacterial translocation occurs and endotoxin spills over, resulting in bacteremia and intestinal endotoxemia [48, 49]. The more severe the portal hypertension, the worse the inflammatory response, which leads to increased production of pro-inflammatory cytokines (mainly tumor necrosis factor alpha and interleukin-6) [48, 49] and vasodilator factors, especially nitric oxide (NO) in the splanchnic area [50, 51]. This response leads to vasodilatation of the splanchnic arterial vessels. In addition, formation of new vessels in the mesenteric circulation occurs with progression of cirrhosis [52–54]. Because of splanchnic vasodilatation, there is a reduction in systemic vascular resistance and a decrease in the effective circulating blood volume, which in turn activates the renin–angiotensin–aldosterone system (RAAS). This potentially unloads the high-pressure baroreceptors in the carotid body and aortic arch and activates the sympathetic nervous system (SNS) to non-osmotic release of vasopressin [52, 55]. These changes lead to intense renal vasoconstriction and reduced GFR [56–58]. With worsening of the liver disease and progression of cirrhosis, further splanchnic vasodilatation occurs, creating a vicious cycle that favors further activation of the RAAS and SNS and vasopressin release, and subsequent intensification of renal vasoconstriction [59].

### Exaggerated inflammatory response

Increased cytokine production upregulates NO synthase leading to increased production of NO and ultimately to exaggerated splanchnic vasodilation [50]. Injury to hepatocytes is known to produce DAMP (damage-associated molecular pattern) molecules, such as high-mobility group box-1 (HMGB1) proteins, which further increase the inflammatory response [13, 26]

### Renal blood flow and autoregulation in HRS

Renal autoregulation maintains a constant renal blood flow under normal conditions. However, in cirrhosis, due to sympathetic stimulation and the exaggerated vasoconstrictor state, the autoregulation becomes disturbed, resulting in lower renal blood flow which becomes blood pressure dependent [56–58].

### Cardiac

Another very important contribution to the development of HRS comes from cardiac and hemodynamic affection. Cardiac dysfunction may remain subclinical, but it becomes unmasked in certain clinical situations that involve stress, such as large volume paracentesis without adequate plasma volume replacement. Though hyperdynamic circulation is the hallmark of liver failure, there is evidence that in advanced liver disease there might be a decline in cardiac performance, ultimately leading to renal hypoperfusion. The development of cirrhotic cardiomyopathy in adults is an important contributing factor in HRS. However, its contribution to HRS in the pediatric patient population is not clear. In cirrhotic cardiomyopathy, both systolic and diastolic contractile response to stress is reduced. In addition, there is evidence of ventricular hypertrophy or dilatation that results in decreased ejection fraction and reduced renal perfusion following any stressful stimulus, setting the stage for kidney injury leading to HRS [60–62].

### Adrenal insufficiency

The term “hepato-adrenal syndrome” was proposed to define adrenal insufficiency in patients with advanced liver disease with sepsis and/or other complications [63]. This leads to vascular hypo-responsiveness and hemodynamic instability potentially affecting renal vasculature and perfusion, thus setting the scene for AKI. Probable causes of adrenal involvement have been postulated to be decreased levels of high-density lipoprotein cholesterol and high levels of pro-inflammatory cytokines and circulatory endotoxin [64].

### Intra-abdominal hypertension

Massive ascites can lead to raised intra-abdominal pressure (IAP) which can compress on the renal vasculature and lead to decreased renal blood flow and a setting of decreased urine output and AKI [65, 66]. We routinely measure IAP in patients with chronic liver disease and ascites with decreased urine output. Therefore, it is vital that ascites is drained and all measures taken to reduce the IAP.

### Biliary salt-induced AKI

Bilirubin can cause a functional proximal tubulopathy or may precipitate into casts, resulting in acute tubular injury. This condition is known as bile cast nephropathy [67]. Bilirubin causes inflammatory and obstructive damage to kidneys, resulting in kidney injury in patients with high bilirubin [68].

**Fig. 2:**
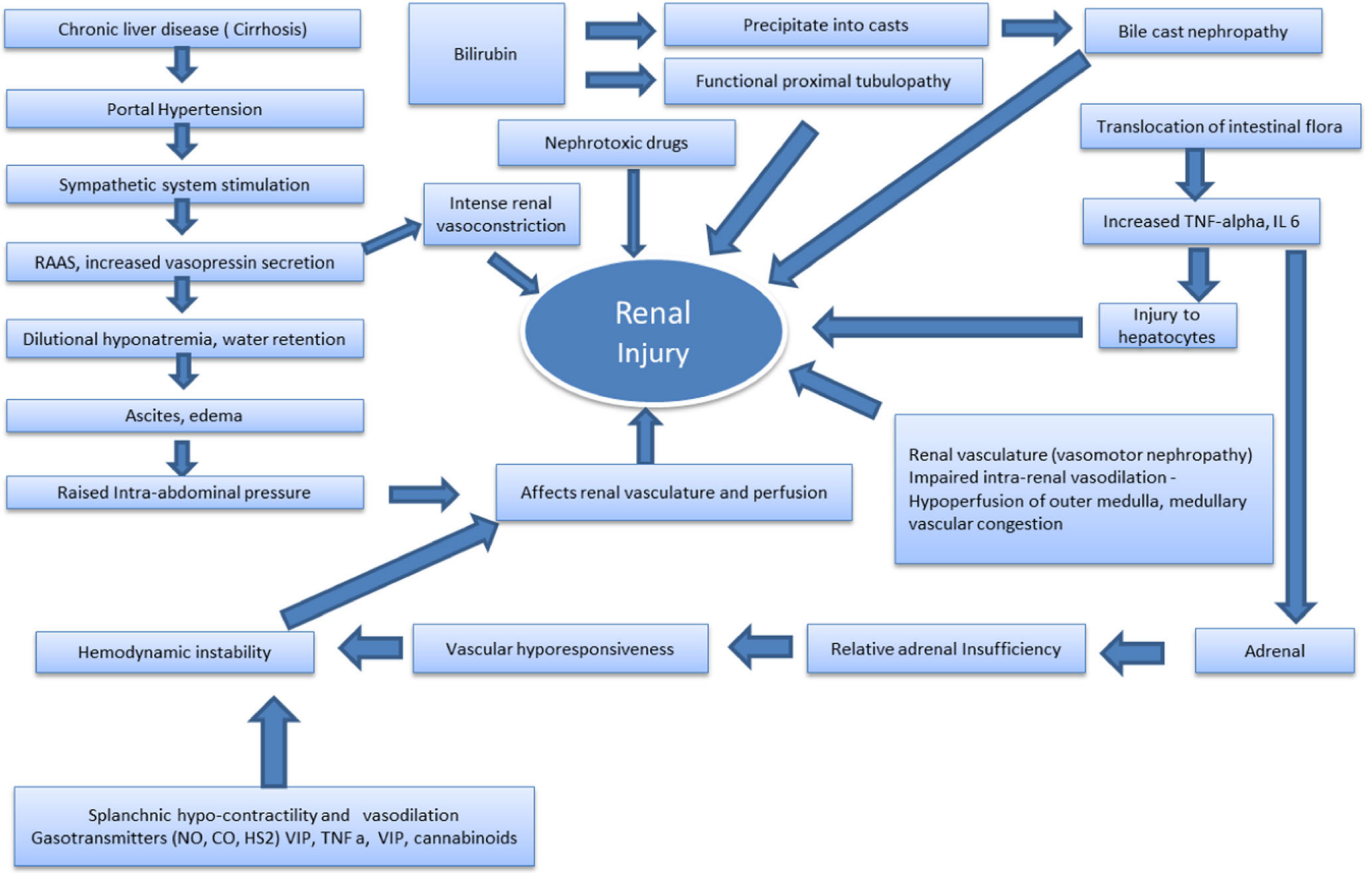
Mechanisms for renal injury in cirrhosis [25, 29, 48, 51, 56, 58, 65–73].* CO* Carbon monoxide,* HS2* hydrogen sulfide,* IL* interleukin,* NO* nitic oxide,* RAAS* renin–angiotensin–aldosterone system,* TNF* tumor necrosis factor,* VIP* vasoactive intestinal peptide

The various pathophysiologic mechanisms of AKI in patients with liver disease are summarized in Fig. 2.

## Diagnosis and role of biomarkers

It is of both prognostic and therapeutic importance that the specific etiology of renal failure in cirrhosis is correctly identified. Various biochemical and clinical criteria have been used historically to differentiate between different causes of AKI in patients with liver disease. However, these criteria fail to accurately differentiate between the various etiologies of AKI. Therefore, to pinpoint accurately the specific etiology of AKI in patients with cirrhosis, recent ICA 2012 guidelines recommend the importance of the use of new urinary biomarkers. Urinary biomarkers of tubular damage which have recently been studied in diagnosing AKI are neutrophil gelatinase-associated lipocalin (NGAL), kidney injury molecule-1 (KIM-1) interleukin-18 (IL-18) and liver fatty acid-binding protein (L-FABP) [25, 74–76]. A recent cohort study (TRIBE–AKI Consortium) in adults used urinary biomarkers (tubular injury markers [NGAL, IL-18, KIM-1, L-FABP], tubular function markers [FeNa] and glomerular injury markers [albumin]) to differentiate between various causes of AKI in liver failure. The authors demonstrated a 13.33-fold higher relative risk (range 4.40–40.39) of having ATN as the cause of AKI if all four biomarkers in the panel were positive (above predefined threshold) as compared to none [30]. Several other studies have demonstrated that these urinary biomarkers are significantly raised in patients with AKI secondary to ATN/ischemic AKI as compared to patients with HRS or pre-renal AKI [30, 75]. However, these findings need to be confirmed in future studies, especially in the pediatric population. In the following two subsections we briefly describe the two most important biomarkers used in clinical practice.

### Neutrophil gelatinase-associated lipocalin

Biochemically, NGAL consists of 178 amino acids and belongs to the lipocalin superfamily [77]. Detection of urinary NGAL occurs due to absorption defects secondary to tubular injury, both proximal and distal [78]. The most important role of a biomarker like NGAL is its ability to detect patients with subclinical AKI before SCr rises [79]. Verna et al. demonstrated that patients with HRS have intermediate urinary NGAL levels. They hypothesized that severe vasoconstriction in the renal vasculature may cause sub-clinical (patchy) tubular epithelial damage which can lead to increased urinary NGAL. They also demonstrated that raised urinary NGAL measurement on hospital admission predicted poor clinical outcome and that a urinary NGAL value of > 110 ng/ml was predictive of inpatient mortality [80].

### Cystatin C

Cystatin C needs special mention in view of its upcoming role in the diagnosis of AKI in liver disease. It is a small protein (13 kDa) which belongs to the cystatin superfamily (cysteine endopeptidase inhibitors). It is produced by all nucleated cells of the body and is released into the blood at a constant rate [81]. It acts as an early marker of glomerular dysfunction [78]. The concentration of cystatin C is independent of age, gender and muscle mass. Tubular damage can result in increased urinary excretion of cystatin C secondary to decreased reabsorption and degradation [82]. A cystatin C level of > 1.23 mg/L is thought to be better at predicting AKI than SCr in adult cirrhotic patients [83]. At our institution, Samyn et al. examined the use of cystatin C as a reliable marker for assessment of renal dysfunction in children with liver disease and after liver transplant [84]. Cystatin C-based GFR formulas can provide an accurate estimation of nuclear GFR in the pediatric population, including transplant recipients [85] and hence should overcome some of the shortcomings of SCr as a biomarker for AKI in liver disease.

## Treatment

Pediatric data on the management of HRS is very scarce, partly due to the small number of patients and partly due to the difficulty in diagnosing this condition. The most up-to-date report is from Debray et al. in 2006 [86]. As with most other diseases, measures to prevent HRS are extremely important in reducing morbidity and mortality. Here we present the treatment algorithm used at our center (based on experience and extrapolated from adult literature) (also refer to Tables 4 and 5).

1. Prophylactic antibiotics: As bacterial translocation is a major precipitant of vasodilator release through mediators like tumor necrosis factor alpha, prophylactic antibiotics prevent bacterial translocation and suppress pro-inflammatory cytokine formation, an important pathogenic factor in HRS [87, 88]. Therefore, we start empiric antibiotic therapy in patients with cirrhosis and AKI.
2. Nephrotoxic drugs: It is important to curtail the use of nephrotoxic drugs in patients with chronic liver disease. The use of nephrotoxic drugs must be avoided, and if they are being used, their need should be reviewed on a regular basis and doses adjusted according to severity of renal dysfunction.
3. Maintain intravascular volume: It is important to ensure that the intravascular status of the patient is maintained. The risk factors for HRS appear to be similar to those for AKI of any other etiology [23, 30, 74, 89]. We recommend early, aggressive treatment of hypovolemia with fluids/blood products using bedside clinical cues alongside the use of cardiac output monitoring (ultrasound or invasive).
4. Paracentesis: As cirrhosis progresses, ascites becomes progressively resistant to diuretics, and with increasing ascites there is raised IAP with attendant adverse effects on renal and systemic hemodynamics [90, 91]. Therefore, ascites should be managed with paracentesis followed by albumin infusion. In cirrhotic patients, RAAS activation causes sodium retention, and the use of aldosterone antagonists (such as spironolactone) have been demonstrated as being effective in the management of ascites [92].

When ascites becomes refractory (ascites that does not respond to sodium restriction and high-dose diuretic treatment and recurs rapidly after therapeutic paracentesis) [35], therapeutic modalities include large volume paracentesis with albumin administration, trans-jugular intra-hepatic porto-systemic shunt (TIPSS) and liver transplantation [93–95]. TIPSS is an effective treatment for refractory ascites, but concerns about increase in encephalopathy and survival benefit make it a less favorable treatment option.

## Pharmacological treatment of HRS

### Albumin

Albumin serves as an efficient plasma expander. Additional benefits of albumin include its role in binding and transporting both exogenous and endogenous substances, anti-inflammatory and antioxidant action, immune-modulatory role and endothelial stabilization [96, 97]. The role of albumin in cirrhosis is secondary to its ability to mobilize ascitic fluid due to its effect on plasma oncotic pressure. Studies have demonstrated the role of albumin in the prevention of circulatory dysfunction after large volume paracentesis [98, 99].

### Vasoconstrictors

Arterial vasodilation is considered to be an important pathophysiological mechanism of HRS; hence, the use of arterial vasoconstrictors can be therapeutic. Vasoconstrictors have the potential to reverse splanchnic vasodilation, increase the circulating blood volume and ultimately reverse renal vasoconstriction and HRS.

Several vasoconstrictors are in use, including vasopressin analogues (terlipressin, ornipressin) midodrine + octreotide and norepinephrine. Terlipressin, is a prodrug that is converted to its active form, lysine vasopressin, after cleavage of three glycyl groups. This cleavage results in a slow release of the vasoactive form. The half-life of terlipressin is 6 h, and since lysine vasopressin is released over a sustained period, it can be administered by bolus injection rather than by continuous infusion [100]. Terlipressin causes vasoconstriction in the arterioles of the splanchnic circulation, decreasing portal flow and thereby redistributing blood flow to the kidneys [101, 102]. Yousef et al. demonstrated an improvement of serum creatinine and urine output after terlipressin use in children [46].

A few institutions use continuous infusion rather than bolus injection as a mode of administration of terlipressin with comparable responses but less severe complications [103, 104]. The rise in blood pressure is more sustained when terlipressin is used as an infusion compared to a bolus, along with a better response in reducing SCr. We use an infusion dose of 10–20 μg/kg/day. It is very difficult to conclude anything meaningful from the scant reports on the use of terlipressin in pediatric patients other than that terlipressin might have a possible role in children with mild-to-moderate HRS-1. It also appears that the dose of terlipressin considered effective for pediatric HRS may be lower than that required in children with refractory septic shock, thus potentially avoiding drug toxicity.

Most of the side effect profile of terlipressin is related to its severe vasoconstrictive properties, which can affect various blood vessels, leading to coronary ischemia, gut ischemia and limb ischemia. Cardiac arrhythmias and cerebrovascular events are other known side effects. However, terlipressin is less likely to cause many of these side effects as compared to vasopressin.

 Various adult studies have shown that combined therapy with terlipressin + albumin has better results in terms of HRS reversal than treatment with terlipressin alone or albumin alone, but it is important to note that not all patients with HRS respond to terlipressin and albumin [105–110]. The most important predictor of response to terlipressin is the baseline SCr level at the time of initiation of terlipressin therapy, i.e. the degree of renal failure. It is postulated that if patients have very high SCr levels at the initiation of terlipressin therapy, response will be limited, and in these cases the risk of severe vasoconstriction versus very little therapeutic response must be balanced. An important controversial point which deserves special mention is the incidence of relapse after vasoconstrictor therapy is stopped.

### Vaptans

Vaptans are V2 receptor antagonists. They are aquaretic agents that promote water excretion and diuresis with dilute urine and improve hyponatremia. They also block V2-mediated vasodilatation. Moreover, V2 receptor antagonism increases plasma vasopressin concentrations, which may cause unopposed hyper-stimulation of the vasoconstrictor V1 receptors, leading to free water excretion. This action results in improvement of hyponatremia and therefore improved ascites. The hemodynamic effects of splanchnic vasoconstriction present a particularly attractive theoretical treatment option in these patients [110, 111]. Recent studies show that vaptans could play a role in elevating serum sodium concentration in cirrhotic patients [111].

## Extracorporeal therapies

### Renal replacement therapy

Continuous rather than intermittent hemodialysis (HD) is the preferred mode of dialysis in these hemodynamically sick patients, particularly those who are listed for liver transplantation and have failed to respond to medical therapy, or those who have suffered an acute reversible precipitating event. Continuous renal replacement therapy (RRT) expedites the removal of solutes such as ammonia and lactate, helps fluid and electrolyte balance and creates space for nutrition in these patients who are in a catabolic state. If the liver becomes available before multiple organ failure becomes refractory, continuous RRT can serve as a bridge to liver transplantation, but it should be considered at an early stage to help prevent further deterioration before liver transplantation becomes available [112]. RRT is especially indicated in life-threatening complications, such as severe hyperkalemia, intractable metabolic acidosis, fluid overload and other uremic complications [9, 113]. Unfortunately, the response to vasoconstrictor therapy becomes very difficult to assess once a child is started on continuous RRT.

### Liver assist devices and tandem therapies

Extracorporeal liver assist devices (LAD) are being increasingly used in clinical practice. They are temporizing artificial support systems which remove toxins from the circulation. The devices available currently include single-pass albumin dialysis (SPAD), plasma exchange (PE) combined with HD, Prometheus dialysis and the molecular adsorbent recirculating system (MARS) [114, 115]. Simultaneous use of PE and HD, also known as tandem PE and HD (TPH), may be an additional resource for treating patients who may benefit from using both therapies concurrently [116]. Absolute indications in pediatric practice for LAD use are extrapolated from adult literature and include (among other factors) hepatic encephalopathy (equal ≥ grade 3) and coagulation failure [114].

#### Molecular adsorbent recirculating system

The MARS is an extracorporeal system which combines high-flux HD, filtration and adsorption and uses an albumin-enriched dialysate to remove toxins [117, 118]. A recent European, multicentered randomized controlled study (RELIEF trial) showed benefit in renal function at day 4 with the use of MARS as compared to the standard treatment arm [119]. A recent meta-analysis showed that MARS conferred a survival benefit in patients with acute liver failure, but the authors could not find evidence that it improved survival in patients with ACLF [120].

#### Prometheus system

The Prometheus® system (Fresenius Medical Care, Bad Homburg, Germany) is another extracorporeal system which is based on the principles of fractional plasma separation with high-flux HD [117, 121]. Its advantages over MARS are that cytokines, coagulation factors and platelets remain unaltered with its use [122] and a higher reduction of toxins, such as ammonia, urea and bilirubin can be achieved [123]. Currently, a single-center randomized controlled trial is underway with the use of the Prometheus system in adult patients with HRS, which may shed some light on its efficacy [124].

## Surgical options

Trans-jugular intrahepatic portosystemic shunt (TIPSS)- Since increased portal pressures play an important role in the pathogenesis of HRS, surgical modalities to decrease portal hypertension (TIPSS) have a role in the management of HRS [125].

### Liver and Liver–kidney transplantation

In patients who do not respond to any treatment modality, liver transplant is the only option. Though many children with kidney disease recover after liver transplant, a proportion continue to require RRT. Currently, the focus is gradually shifting from liver transplantation to simultaneous liver–kidney transplant. It is an accepted strategy in patients with both ACLF and end-stage renal disease [126]. There are now well-established criteria for simultaneous liver and kidney transplantation in patients awaiting liver transplantation who develop HRS [127]. However, the prognosis of children who develop HRS and require continuous RRT for a prolonged period is almost universally poor.

A summary of the various treatment options in patients who develop AKI in liver disease are is presented in Table 4.

**Table 4:** Proposed algorithm for management of children with liver disease and concomitant acute kidney injury

Treatment options in patients with AKI in liver disease
Treat associated conditions: • GI bleeding/hypovolemia: fluid resuscitation • Infections: aggressive antibiotics (as per local antibiogram) • Adrenal insufficiency • Avoid nephrotoxic drugs • Treat raised IAP (drain and replace with albumin) • Large volume ascites: paracentesis • Differentiate between natural progression of liver disease with its complications vs. acute AKI with other organ dysfunction • Once in ICU: cardiac output monitoring, fluids, full organ support, prioritize transplant listing • Early vasoconstrictors and albumin
Pharmacological therapy: • Albumin • Vasoconstrictors including vasopressin and vasopressin analogues, octreotide, norepinephrine • Vaptans (rarely used)
Assist devices: • Continuous renal replacement therapy ± plasmapheresis • MARS • SPAD • Prometheus
Surgical therapy • TIPSS ((very rarely done) • Liver transplant

GI, gastrointestinal; IAP, intra-abdominal pressure; TIPSS, trans-jugular Intrahepatic Porto-systemic shunt; ICU, intensive care unit, MARS, molecular adsorbent recirculation system; SPAD, single pass albumin dialysis

## Proposed algorithms for managing patients with AKI in chronic liver disease

When confronted with a child with cirrhosis and AKI, the following basic principles apply: treat infections aggressively, actively reduce IAP by paracentesis, resuscitate with fluid to prevent renal hypoperfusion and start treatment with vasoconstrictors early together with albumin. Though there is good evidence to use combined terlipressin + albumin, we start with noradrenaline as it is a more commonly used vasopressor in any pediatric critical care setting with good familiarity among critical care staff. If there is no response to noradrenaline at a dose of 0.5–1 mcg/kg/min, we recommend starting terlipressin infusion at a dose of 10 mcg/kg/day, very carefully looking for side effects especially microvascular ischemia. If there is no response to terlipressin in the form of decreasing SCr or sustained blood pressure increment, the dose of terlipressin can be doubled at 48 h. If there is still no response by 4–5 days, early RRT needs to be started. Liver transplant is the ultimate treatment, but if HRS is still present, these patients have a poor prognosis even after liver transplant. It is very challenging to list a patient with HRS for liver transplantation, and a multidisciplinary team needs to discuss the prognosis following liver transplantation. This approach is summarized in Table 5.

**Table 5:**
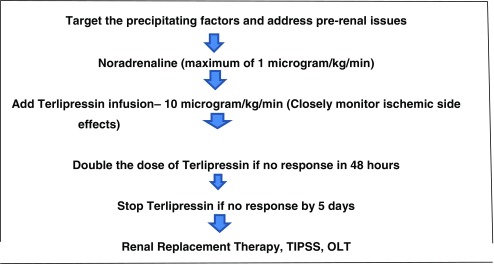
Personal practice to management of hepatorenal syndrome

TIPSS: Trans-jugular Intrahepatic Porto-systemic shunt, OLT: Orthotopic Liver Transplant

## Future directions

Two questions for the future are:

1. Are there any new pharmacological or non-pharmacological treatments available or currently being researched for the treatment of HRS?
2. Awaiting the availability and accessibility to new therapies, can we do anything to improve the prognosis of these patients who do not seem to respond to standard therapy?

In the treatment of AKI in liver disease, it is important to stick to the basics. It is equally important to accurately differentiate HRS from other types of AKI, especially ATN. Some treatment failures are wrongly attributed to terlipressin inefficacy, but these in fact are due to the use of this drug in incorrectly diagnosed HRS. Therefore, the role of urine biomarkers, particularly NGAL and IL-18, can potentially help in the differential diagnosis between HRS and ATN. If the role of urinary biomarkers in the differential diagnosis of various etiologies of AKI in liver disease is confirmed in larger studies, it will not be surprising to see their incorporation in the diagnostic algorithm of HRS-1 [12, 30, 80]. In addition to accurate diagnosis, some drugs under investigation need a special mention. Serelaxin is a recombinant form of the human peptide relaxin-2 that has been shown to be a renal vasodilator in healthy volunteers. Serelaxin selectively and very effectively increases renal blood flow with no alterations in mean arterial pressure [128]. If researchers could replicate the effect of selective renal vasodilation in patients with renal failure, serelaxin could prove an invaluable drug in the treatment of HRS-1 where renal vasoconstriction plays an important role in the pathogenesis.

There is a very important role of inflammation in ACLF where HRS occurs as a part of multi-organ failure. Here failure of one organ system can affect the function of another organ system. Therefore, one needs to target not only the systemic vasodilation, which plays an important role in the pathogenesis of HRS, but also the systemic inflammation and to optimize the function of other organs. Removal of inflammatory and vasoactive mediators by the use of plasmapheresis could be a useful therapeutic adjunct therapy [129]. In a recent study on acute liver failure in adults, total PE decreased the systemic inflammatory response and improved organ failures and survival. Innate immunity is depressed in ACLF. Stimulation of this immunity could lead to improved liver function, prevent the occurrence of multi-organ failure, including HRS, and potentially improve survival. The role of granulocyte colony-stimulating factors could be another promising approach in achieving the stimulation of the innate immunity [130].

## Conclusion

Though AKI is common in cirrhotic patients with ascites, not all patients of cirrhosis who develop AKI have HRS. Criteria to diagnose AKI in liver patients are different from those used to diagnose AKI in patients with non-liver diagnoses, but unfortunately there are no criteria specific to pediatric patients. Though diagnostic criteria exist to diagnose HRS, differentiating it from other causes of AKI in cirrhotic patients continues to be a challenging task in some cases. Biomarkers may start to play an important role in differentiating between the various causes of AKI in this patient population, which is crucial for diagnostic, therapeutic and prognostic purposes. Therefore, it will not be surprising to see the incorporation of biomarkers in the definition of AKI in patients with liver disease soon (instead of creatinine). Vasoconstrictors seem to play an important role in the treatment of HRS if they are started early. The role of prolonged vasoconstrictors to bridge these patients to liver transplant is open to debate and future trials.

## Key learning points

- Criteria to diagnose AKI in cirrhosis are not the same as those to diagnose non-liver AKI; no formal criteria exist in pediatrics.
- Not every AKI in cirrhosis is HRS; biomarkers could potentially help to differentiate the various causes of AKI.
- HRS is extremely rare in the pediatric patient population and carries a very poor prognosis.
- Prevention of precipitating factors is important.
- Vasoconstrictors seem to have a role in management of patients with AKI

## Multiple-choice questions (answers are provided following the reference list)

1. Which one is the least common cause of AKI in patients with liver disease?
  - Hypovolemia
  - ATN
  - Infections
  - HRS
2. A terlipressin responder is one who has:
  - Pre-renal AKI
  - Severe HRS with SCr > 7 mg/dl
  - Early increase in mean blood pressure followed by return to baseline
  - Early onset AKI with modest increase in SCr and sustained increase in blood pressure
3. Which one of the following statements is NOT true about AKI in liver disease?
  - Definition of AKI in liver and non-liver disease patients is different
  - SCr can be reliably used to diagnose AKI in these patients
  - Biomarkers help to differentiate ATN from HRS
  - Cystatin C can be a useful adjunct in diagnosis of AKI
4. The KDIGO definition differs from the IAC 2012 definition with the following exception?
  - Use of SCr as a marker
  - Utilization of continuous RRT
  - Defines response to treatment
  - Definition of what constitutes the baseline SCr values
5. Which of the following is the initial management of HRS?
  - Prometheus system
  - TIPSS
  - MARS
  - Treat associated conditions, volume expansion and vasoconstrictors
